# Percutaneous tracheostomy: it’s time for a shared approach!

**DOI:** 10.1186/cc13974

**Published:** 2014-07-07

**Authors:** Maria Vargas, Paolo Pelosi, Giuseppe Servillo

**Affiliations:** 1 Department of Neurosciences, Reproductive and Odontosthomatological Sciences, University of Naples “Federico II”, Naples, Via Pansini 16 – 80100, Naples, Italy; 2 Department of Surgical Sciences and Integrated Diagnostics-IRCCS San Martino IST, University of Genoa, L.go R. Benzi 16-1632, Genoa, Italy

## 

In a previous issue of *Critical Care*, Simon and colleagues [1] reported the incidence of death related to percutaneous tracheostomy (PT). Fatal complications occurred in 31% of cases during the procedure and in 49% of cases within the first week of the tracheostomy [1]. In a later issue of *Critical Care*, Rajendran and Hutchinson [2] suggested the use of a checklist, adapted from the World Health Organization (WHO) surgical safety checklist, to improve safety and reduce errors and harm related to the PT procedure in critical care. However, a recent observational study performed in 101 hospitals in Ontario, Canada, did not find any reduction in mortality or complications after the implementation of the WHO checklist in more than 100,000 surgical procedures [3].

PT is widely used in critical care, although no clinical guidelines have been developed to suggest the best practice for this invasive and risky procedure. Surveys on PT, performed in different European countries, have shown the presence of a shared clinical practice [4]. We think that, lacking clinical guidelines to provide the best available scientific evidence and to reduce inappropriate variation in PT practice, a careful analysis of different surveys may suggest to physicians the most common practice associated with PT. Table 1 shows shared clinical practice for PT from an analysis of seven national surveys performed in France (where 152 intensive care units participated in the survey), Germany (505), Italy (130), The Netherlands (63), Spain (100), Switzerland (48), and the UK (197).

**Table 1:** Shared clinical practice for percutaneous tracheostomy from an analysis of seven national surveys in Europe

Findings	Most common practice
Indications	Long-term mechanical ventilation, weaning failure, and upper airway obstruction
Techniques	Ciaglia single dilator and guide-wire dilating forceps
Timing	7 to 15 days after intensive care unit admission
Involved physicians in percutaneous tracheostomy	Intensivists; ear, nose, throat specialist; and general surgeon
Neck ultrasound evaluation	Screening before the procedure to assess at-risk structure
Ventilation protocol	Largely used with volume-controlled ventilation
Sedation protocol	Largely used in association with local anesthesia, analgesia, and neuromuscular blocking
Airway management	Endotracheal tube in place
Fiberoptic bronchoscopy	Largely used
Diameter of fiberoptic bronchoscope	3 to 5 mm
Intraprocedural complications	Minor bleeding

The analysis was of seven national surveys performed in France (where 152 intensive care units participated in the survey), Germany (505), Italy (130), The Netherlands (63), Spain (100), Switzerland (48), and the UK (197).

## Abbreviations

PT: percutaneous tracheostomy; WHO: World Health Organization.

## Competing interests

The authors declare that they have no competing interests.
